# HDAC5, a potential therapeutic target and prognostic biomarker, promotes proliferation, invasion and migration in human breast cancer

**DOI:** 10.18632/oncotarget.9274

**Published:** 2016-05-10

**Authors:** Anqi Li, Zebing Liu, Ming Li, Shuling Zhou, Yan Xu, Yaoxing Xiao, Wentao Yang

**Affiliations:** ^1^ Department of Pathology, Fudan University Shanghai Cancer Center, Department of Oncology, Shanghai Medical College, Fudan University, Shanghai, P.R. China; ^2^ Department of Pathology, Renji Hospital, School of Medicine, Shanghai Jiaotong University, Shanghai, P.R. China

**Keywords:** histone deacetylase, breast cancer, HDAC inhibitor, proteasome inhibitor, drug target

## Abstract

**Purpose:**

Histone deacetylase 5 (HDAC5) is an important protein in neural and cardiac diseases and a potential drug target. However, little is known regarding the specific role of HDAC5 in breast cancer (BC). We aimed to evaluate HDAC5 expression in human breast tumors and to determine the effects of the inhibition of HDAC5 expression in BC cells.

**Experimental design:**

HDAC5 expression was evaluated in BC patients and was correlated with clinical features and with patient prognosis. Functional experiments were performed using shRNA and the selective HDAC inhibitor LMK-235 for HDAC5 knockdown and inhibition in BC cells. The synergistic effects of LMK-235 with the proteasome inhibitor bortezomib were also examined.

**Results:**

HDAC5 was extensively expressed in human BC tissues, and high HDAC5 expression was associated with an inferior prognosis. Knockdown of HDAC5 inhibited cell proliferation, migration, invasion, and enhanced apoptosis. The HDAC5 inhibitor LMK-235 inhibited cell growth and induced apoptosis, while the inclusion of bortezomib synergistically enhanced the efficacy of LMK-235.

**Conclusions:**

Our findings indicate that HDAC5 is a promising prognostic marker and drug target for BC and that the combination of LMK-235 and bortezomib presents a novel therapeutic strategy for BC.

## INTRODUCTION

Breast cancer (BC) is the most common cancer and the leading cause of cancer-related death in women. Advances in novel adjuvant therapies have markedly decreased BC-related mortality [1]. However, the problems of drug resistance and distant metastasis remain unresolved and have resulted in inadequate patient survival. To address these issues, more effective and less toxic targeted therapies are urgently required to improve the cure rate.

Histone deacetylases (HDACs) are enzymes that function in epigenetic gene regulation through chromatin modification. Among 18 human HDACs, HDAC5 (a class IIa HDAC) has been found to contribute to dynamic activities such as synoviocyte activation [2], neural regeneration and repair [3], and myoblast differentiation [4]. Recent studies have reported the aberrant overexpression of HDAC5 in hepatocellular carcinoma [5] and high-risk medulloblastoma [6], whereas HDAC5 downregulation has been reported in colon cancer [7] and is associated with poor prognosis in lung cancer patients [8]. Moreover, Peixoto et al. demonstrated that HDAC5 maintains pericentric heterochromatin structures in human cancer cells and thus represents a potential anticancer drug target [9]. Subsequently, Hsieh et al. reported that pan-HDAC inhibitors (pan-HDACis) induce BC apoptosis via the upregulation of microRNA (miR)-125a-5p, which post-transcriptionally silences HDAC5 [10].

HDACis are inhibitors that induce apoptosis and cell cycle arrest and that impede metastasis, invasion and angiogenesis in cancer cells [11]; thus, they have emerged as exciting new anticancer agents. However, beyond the potent clinical efficacy of pan-HDACis, unfavorable side effects should not be overlooked. Since the class IIb-selective HDACi ricolinostat has been reported to be well tolerated [12], class-selective HDACis should be considered preferentially. LMK-235 is a novel HDACi that exhibits HDAC isoform selectivity, with a preference for HDAC4 and HDAC5 [13]. LMK-235 displays equipotent HDAC inhibition compared with vorinostat and potent cytotoxicity. In addition, many studies have proposed that HDACis effectively synergize with other diverse anticancer agents that exert antineoplastic effects [14, 15]. Among various synergistic models, the combination of HDACis and the proteasome inhibitor bortezomib has shown undisputed success [16, 17].

Here, we aimed to exploit the potential therapeutic role of HDAC5 in BC. First, we investigated HDAC5 expression patterns and their correlation with clinicopathologic features and prognosis in patients with BC. Second, functional studies were performed to examine the results of HDAC5 loss on tumor biology. Finally, we investigated the activity of the novel HDACi LMK-235 and the combined effects of LMK-235 and bortezomib on BC cells.

## RESULTS

### HDAC5 overexpression promotes BC metastasis and correlates with an inferior prognosis

**Figure 1:**
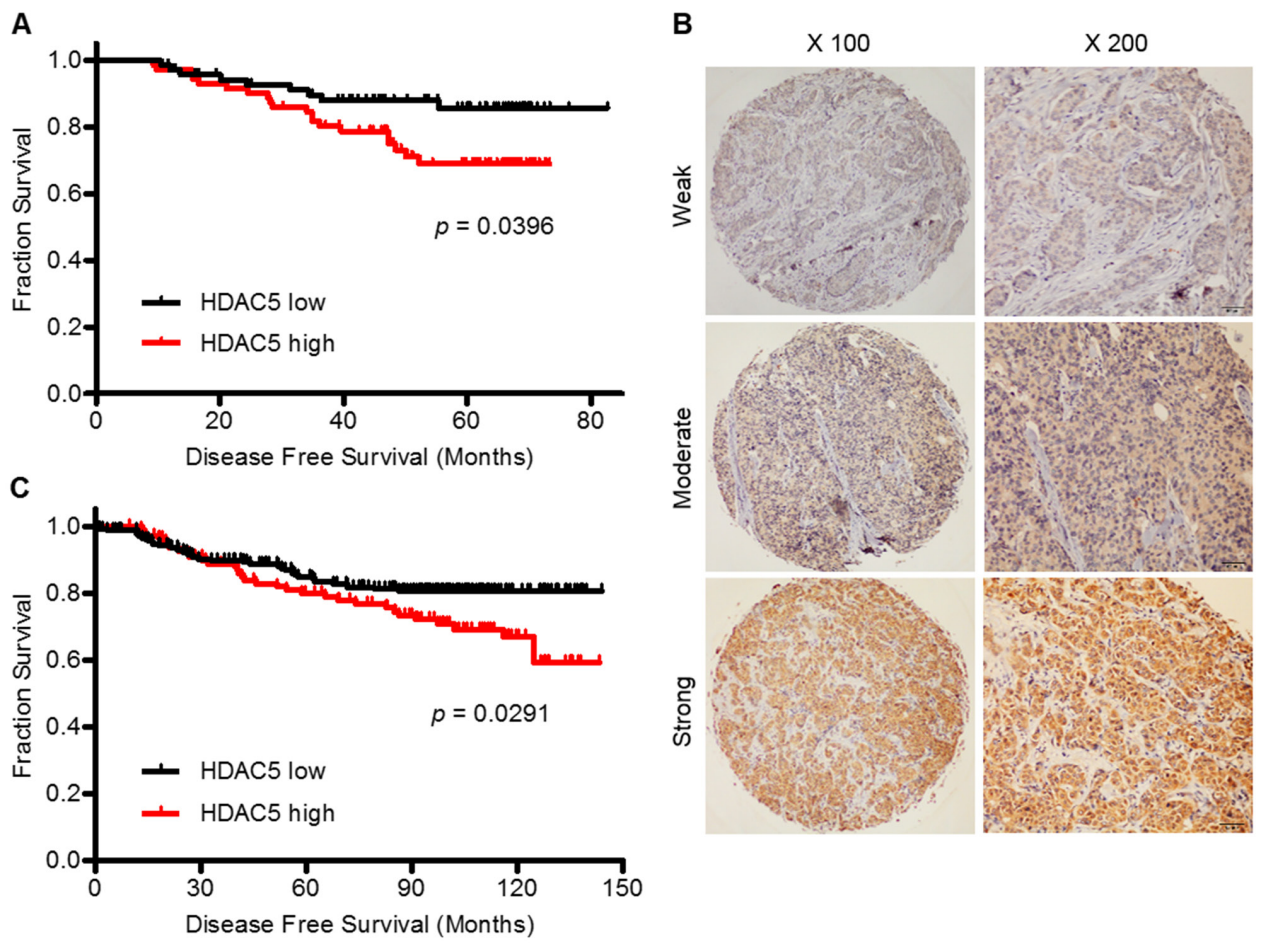
High HDAC5 mRNA and protein expression predict a worse prognosis for patients with BC **A.** Kaplan-Meier curve of DFS for BC patients with low (N=74) and high (N=75) HDAC5 mRNA expression as measured by qPCR. **B.** IHC revealed that HDAC5 protein was expressed widely in BC samples and was expressed predominantly in the cytoplasm of cancer cells. Representative strong, moderate and weak staining is shown. Magnification: ×100, ×200. **C.** Kaplan-Meier curve of DFS for BC patients with low (N=235) and high (N=115) HDAC5 protein expression as measured by IHC. The p value was calculated using the two-sided log-rank test.

To investigate the potential relevance of HDAC5 expression in BC tissues in terms of clinical characteristics, HDAC5 mRNA expression was examined in 149 breast tumor samples. The median patient age was 51 years (range, 30-82 years). Quantitative real-time PCR (qPCR) revealed that 75 (50.3%) patients had high HDAC5 mRNA expression. Clinical and histopathologic characteristics grouped by HDAC5 mRNA expression level are summarized in Supplementary Table S1. High HDAC5 mRNA expression was significantly associated with distant metastasis (*p*<0.028) and molecular subtype (*p*=0.009). Next, we assessed the prognostic value of HDAC5 mRNA expression in terms of disease-free survival (DFS) and overall survival (OS). During a median follow-up of 51.2 months (range, 4.6-82.7 months), 23 patients (15.4%) experienced metastatic relapse, and 10 (6.7%) deaths occurred. Multivariate Cox regression analysis showed that HDAC5 expression was an independent factor associated with DFS (HR=2.33; 95% CI: 1.00-5.30; *p*=0.04) and lymph node (LN) status (HR=1.37; 95% CI: 1.00-1.87; *p*=0.047; Supplementary Table S2). The Kaplan-Meier survival curve demonstrated that HDAC5 overexpression was correlated with a significant decrease in DFS (HR=2.148; 95% CI: 1.037-4.449; *p*=0.0396; Figure 1A), whereas no significant difference was observed in OS (HR=0.9480; 95% CI: 0.287-3.129; *p*=0.9295).

In addition, we performed immunohistochemistry (IHC) to evaluate HDAC5 protein expression on tissue microarrays (TMA) containing 450 breast tumor samples. In all, 350 samples were available for observation. HDAC5 was predominantly expressed in the cytoplasm of malignant epithelial cells (Figure 1B). A score of >190 was defined as the cut-off point based on the receiver operating characteristic (ROC) curve analysis. Thus, the patients were divided into two groups according to HDAC5 protein expression: 235 (67%) with low expression and 115 (33%) with high expression. The clinicopathological features that correlated with HDAC5 protein expression are presented in Supplementary Table S3. High HDAC5 protein expression was significantly associated with metastasis (*p*=0.001), which was consistent with the mRNA results. The prognostic value of HDAC5 protein expression in terms of DFS and OS was similar to that of HDAC5 mRNA expression for the 350 patients. During a median follow-up of 95.4 months (range, 2.1-143.9 months), 59 patients (16.9%) experienced metastatic relapse, and 34 patients (9.7%) died from disease progression. The multivariate prognostic analysis and the Kaplan-Meier survival curve for this cohort were concordant with those for the qPCR cohort. HDAC5 protein expression (HR=1.94; 95% CI: 1.23-3.05; *p*=0.004) and LN status (HR=1.41; 95% CI: 1.15-1.74; *p*=0.001) were independent factors for DFS (Supplementary Table S4). High expression of HDAC5 at the protein level correlated with a significant decrease in DFS (HR=1.72; 95% CI: 1.057-3.796; *p*=0.029; Figure 1C), whereas no correlation was observed with OS (HR=1.50; 95% CI: 0.731-3.078; *p*=0.269).

### Knockdown of HDAC5 decreases cell proliferation and induces apoptosis in BC cell lines

**Figure 2:**
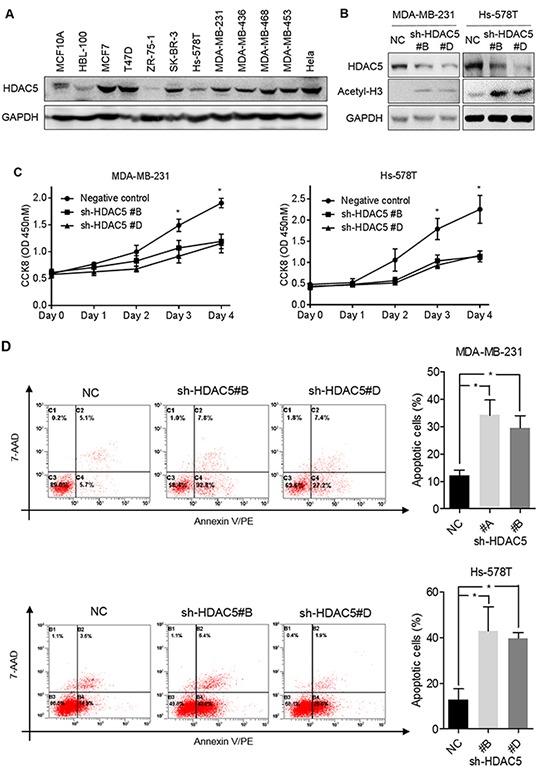
Knockdown HDAC5 decreases cell proliferation and induces apoptosis **A.** Basal HDAC5 expression in 11 breast cell lines was evaluated by western blot analysis. **B.** MDA-MB-231 and Hs-578T cells were transfected with sh-HDAC5 (sh-HDAC5#B or sh-HDAC5#D) or negative control (NC) shRNA. Seventy-two hours after transfection, the levels of HDAC5 and acetyl-histone H3 were examined by western blot. (A and B) GAPDH was used as a loading control. **C.** MDA-MB-231 and Hs-578T cells were incubated for 4 days after transfection with sh-HDAC5#B and shHDAC5#D. Cell growth was evaluated by CCK8 assay. Points indicate the mean of at least three independent experiments. Bars, standard deviation (SD). *p<0.05 between cell lines. **D.** Seventy-two hours after transfection with sh-HDAC5#B, shHDAC5#D and negative control (NC) shRNA, MDA-MB-231 and Hs-578T cells were stained by annexin V/PE-7AAD and measured by flow cytometry for apoptosis. Representative results are shown. The data are presented as the mean ±SD of at least three independent experiments. *p<0.05.

To determine the functional effects of HDAC5 on the biological behaviors of breast cancer cells, basal HDAC5 protein expression was evaluated in eleven breast epithelial cell lines; HeLa cells served as a positive control. As shown in Figure 2A, HDAC5 protein expression was relatively higher in BC cells than in the two normal breast cell lines. MDA-MB-231 and Hs-578T cells were transiently transfected with shRNA against HDAC5, and HDAC5 downregulation was confirmed by western blot analysis (Figure 2B). On the contrary, the levels of acetyl-histone H3 were increased after knockdown of HDAC5 in both MDA-MB-231 and Hs-578T cells (Figure 2B). As shown in Figure 2C, the knockdown of HDAC5 significantly decreased cell proliferation. In addition, flow cytometry showed that HDAC5 deficiency induced early apoptosis in both cell lines (Figure 2D).

### HDAC5 deficiency impedes BC cell migration and invasion

**Figure 3:**
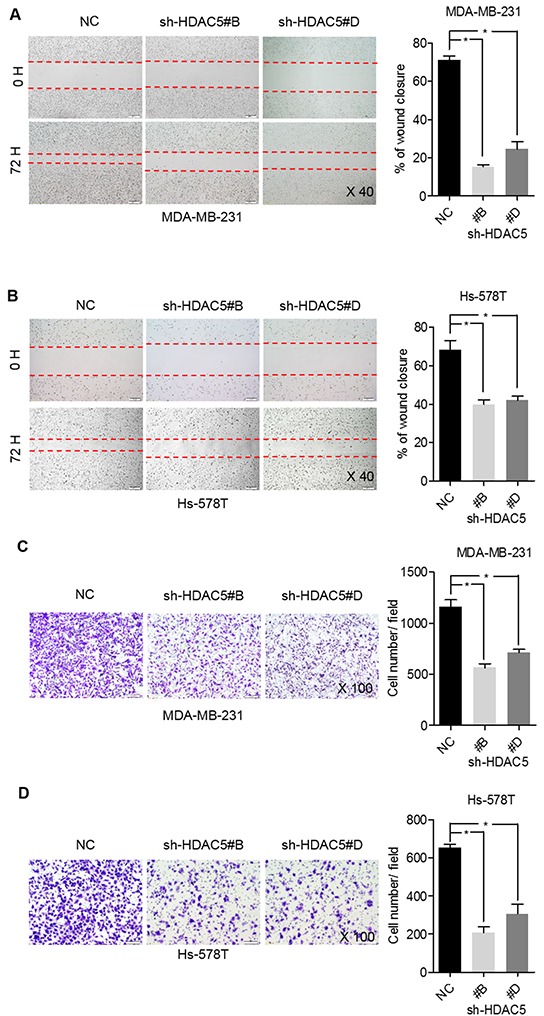
HDAC5 deficiency impedes BC cell migration and invasion **A.** and **B.** MDA-MB-231 and Hs-578T cell motility was assessed by scratch wound-healing assay after transfection with sh-HDAC5#B, sh-HDAC5 #D and negative control (NC) shRNA for 72 hours. Representative results are shown. Magnification, ×40; Columns, mean ±SD of three independent experiments. *p<0.05. **C.** and **D.** Seventy-two hours after transfection with sh-HDAC5#B, shHDAC5#D and negative control (NC) shRNA, the invasiveness of MDA-MB-231 and Hs-578T was assessed using Matrigel invasion chambers. Three separate experiments were conducted, and representative results are shown. Magnification, ×100. Columns indicate the average number of invading cells from 5 random microscopic fields. *p<0.05.

Wound-healing assays demonstrated that HDAC5 knockdown significantly inhibited MDA-MB-231 and Hs-578T cell migration by ~58% and ~30%, respectively (Figure 3A and 3B). Moreover, HDAC5 deficiency markedly inhibited the invasiveness of MDA-MB-231 and Hs-578T cells (~57% and ~73%, respectively; Figure 3C and 3D).

### LMK-235 inhibits proliferation and induces apoptosis of BC cells

**Figure 4:**
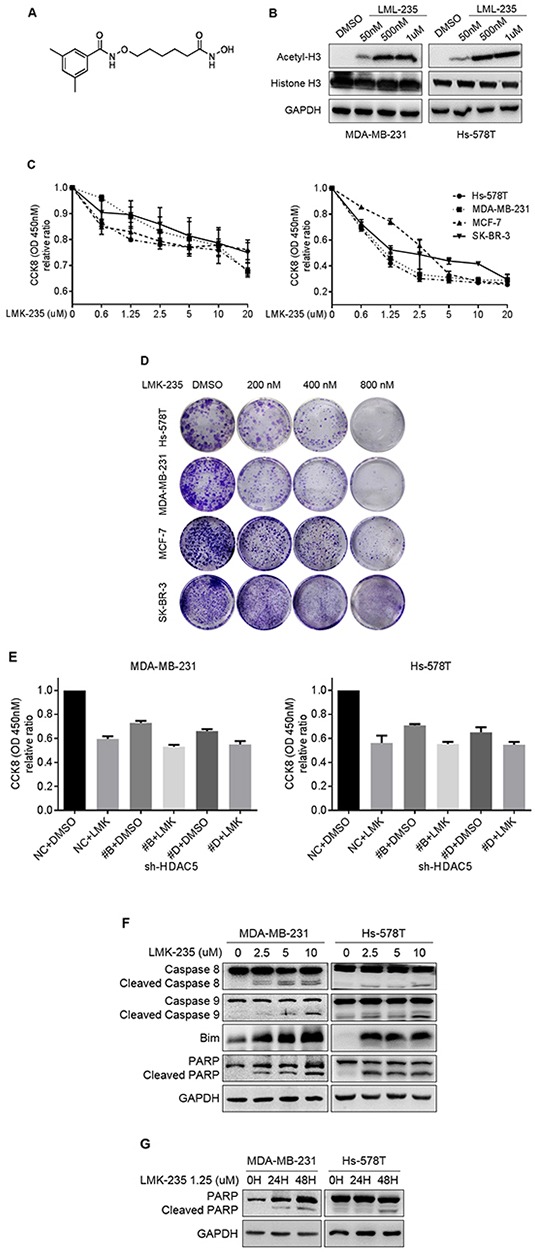
LMK-235 inhibits BC cell proliferation and induces apoptosis **A.** Structure of the class IIa HDACi LMK-235. **B.** MDA-MB-231 and Hs-578T cells were treated with DMSO or 50 nM, 500 nM, or 1 μM LMK-235 for 24 hours. The levels of acetyl-histone H3 and total histone H3 were examined by western blot. GAPDH was used as a loading control. **C.** Hs-578T, MDA-MB-231, MCF-7 and SK-BR-3 cells were treated with LMK-235 (0 to 20 μM) for 24 (left) or 48 (right) hours. Cell proliferation was determined by CCK8 assay. Points indicate the mean of at least three independent experiments. Bars, SD. **D.** Hs-578T, MDA-MB-231, MCF-7 and SK-BR-3 cells were cultured in 12-well plates and treated with the indicated concentration of LMK-235; survival was measured by clonogenic assay. **E.** HDAC5 knockdown cells were treated with 1.25 μM LMK-235 for 48 hours. Cell proliferation was determined by CCK8 assay. Columns, mean ±SD of three independent experiments. **F.** MDA-MB-231 and Hs-578T cells were treated with the indicated concentration of LMK-235 for 24 hours and immunoblotted with anti-caspase 8, anti-caspase 9, anti-Bim and anti-PARP antibodies. **G.** MDA-MB-231 and Hs-578T cells were treated with 1.25 μM LMK-235 for 24 or 48 hours and immunoblotted with an anti-PARP antibody. (F and G) GAPDH was used as a loading control.

The effects of HDAC5 inhibition were examined using the HDACi LMK-235. The HDAC specificity of LMK-235 was validated by measuring its effect on the level of acetyl-histone H3. As shown in Figure 4B, increased concentrations of LMK-235 induced the accumulation of acetyl-histone H3. MDA-MB-231, Hs-578T, SK-BR-3, and MCF-7 cells were exposed to increasing concentrations of LMK-235 (0, 0.625, 1.25, 2.5, 5, 10, and 20 μM; Figure 4C) for 24 and 48 hours. The relative proliferation of the cell lines decreased in a dose- and time-dependent manner. Cell viability was severely compromised after 48 hours of treatment with minimal doses of LMK-235. Long-term clonogenic assays confirmed the growth inhibition induced by LMK-235 at lower concentrations of 0 to 800 nM (Figure 4D). In order to examine the effects of HDAC5 in response to LMK-235, HDAC5 knockdown cells were treated with 1.25 μM LMK-235 for 48 hours. In the presence of LMK-235, the relative proliferation of HDAC5 knockdown cells was further decreased compared with the non-treated knockdown cells (*p*<0.05; Figure 4E). However, no significant difference was observed between negative control cells and HDAC5 knockdown cells in the presence of LMK-235 (*p*>0.05; Figure 4E). Furthermore, the inhibition of proliferation by LMK-235 occurred in a dose- and time-dependent manner along with the increased protein expression of the apoptosis markers Bim, caspase 8 and caspase 9, as well as PARP cleavage (Figure 4F and 4G). The data from flow cytometric analyses of the effects of LMK-235 on apoptosis are not shown.

### Knockdown of HDAC5 enhances the cytotoxic effects of bortezomib

**Figure 5:**
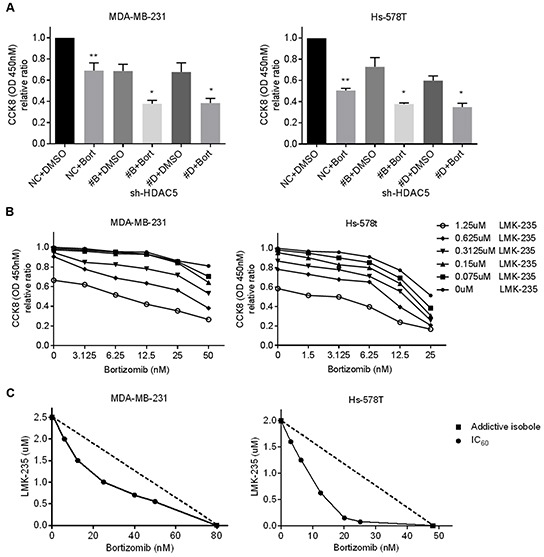

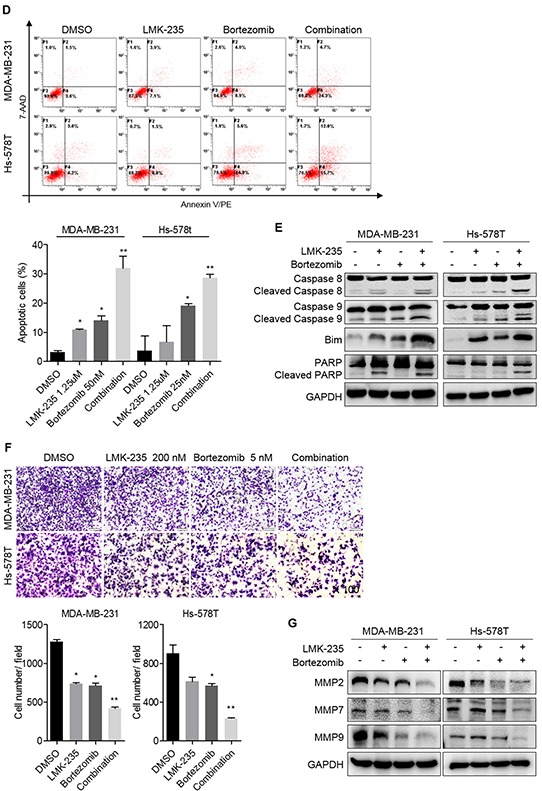
LMK-235 synergizes with bortezomib in BC cells **A.** MDA-MB-231 and Hs-578T cells were treated 50 nM bortezomib or DMSO for 48 hours after transfection with sh-HDAC5#B and sh-HDAC5#D. Cell growth was evaluated by CCK8 assay. Columns, mean ±SD of three independent experiments. **p<0.05 compared with non-treated negative control cells. *p<0.05 compared with treated knockdown cells. **B.** MDA-MB-231 and Hs-578T cells were treated with various combinations of LMK-235 and bortezomib for 48 hours. Cell viability was determined by CCK8 assay. Points indicate the mean of three independent experiments. **C.** Synergism of proliferation inhibition of MDA-MB-231 and Hs-578T cells was analyzed by isobologram analysis. D, E. and G. MDA-MB-231 and Hs-578T cells were treated with 1.25 μM LMK-235 and/or 50 nM bortezomib or with 1.25 μM LMK-235 and/or 25 nM bortezomib for 24 hours. **D.** Apoptosis was assessed by annexin V/PE-7AAD staining and flow cytometry; representative results are shown. Columns represent the mean ±SD of at least three independent experiments. *p<0.05 compared with the DMSO group; **p<0.05 compared with the equivalent doses in the LMK-235- or bortezomib-treated groups. **E.** Cell lysates were immunoblotted with anti-caspase 8, anti-caspase 9, anti-Bim and anti-PARP. GAPDH was used as a loading control. **F.** MDA-MB-231 and Hs-578T cells were plated in Matrigel invasion chambers and treated with 200 nM LMK-235 and/or 5 nM bortezomib for 24 hours. Three separate experiments were conducted, and representative results are shown. Magnification, ×100. Columns indicate the average number of invading cells from 5 random microscopic fields. *p<0.05 compared with the DMSO group; **p<0.05 compared with the equivalent doses in the LMK-235- or bortezomib-treated groups. **G.** Cell lysates were immunoblotted with anti-MMP2, anti-MMP7 and anti-MMP9 antibodies. GAPDH was used as a loading control.

HDAC5 knockdown cells and negative control cells were treated with 50 nM bortezomib for 48 hours. Compared with non-treated cells, bortezomib inhibited cell proliferation of negative control MDA-MB-231 and Hs-578T cells by ~36% and ~50%, respectively (*p*<0.05; Figure 5A). Moreover, the knockdown of HDAC5 further enhanced the cytotoxicity of bortezomib compared with the treated negative control cells and the non-treated HDAC5 knockdown cells (*p*<0.05).

### LMK-235 synergizes with bortezomib in BC cell lines

MDA-MB-231 and Hs578T cells were treated with a single drug or with combinations of various concentrations of LMK-235 and bortezomib for 48 hours. As shown in Figure 5B, the combination of LMK-235 and bortezomib yielded stronger cytotoxic effects compared with either drug alone. The synergism between LMK-235 and bortezomib was analyzed by isobolograms of IC_60_ (Figure 5C). The isoboles lie to the left of the additive isoboles, which indicated synergistic action. The combination indices further demonstrated the synergistic cytotoxicity (Supplementary Table S5). Furthermore, the combined treatments exhibited strong effects on the induction of apoptosis, as determined by flow cytometry and western blot in both cell lines after 24 hours of treatment (Figure 5D and 5E). In addition, MDA-MB-231 and Hs-578T cells were seeded in Matrigel invasion chambers and treated with low nanomolar concentrations of LMK-235 and/or bortezomib to prevent the cytotoxic effects on cell growth. Surprisingly, we found that the combination treatment significantly impeded cell migration (Figure 5F). The expression levels of MMP2, MMP7, and MMP9 were also downregulated by the combination treatment (Figure 5G).

## DISCUSSION

The role of HDAC5 in tumorigenesis and cancer progression is controversial [7–8]. The present study shows that HDAC5 mRNA and protein were both widely expressed in breast tumor tissues and that the relatively high expression of HDAC5 was associated with an inferior prognosis in patients with BC. In addition, functional studies confirmed the oncogenic effects of HDAC5 in BC, which are consistent with the findings of Hsieh et al. [10].

Notably, HDAC5 (a class IIa HDAC) shuttles between the nucleus and the cytoplasm in response to certain cellular signals. Milde et al. reported that cellular HDAC5 staining in primary medulloblastoma was predominantly nuclear [6]. However, in our study, HDAC5 was primarily localized in the cytoplasm in BC cells. Nuclear-cytoplasmic trafficking of HDAC5 has been reported to be involved in the cellular differentiation that occurs during muscle and axon regeneration [3, 4]. In carcinogenesis, the expression patterns and underlying mechanism of nuclear-cytoplasmic shuttling of HDAC5 warrant further investigation.

HDAC5 and HDAC9 are valuable markers for medulloblastoma risk stratification and are potential novel drug targets [6]. However, specific HDAC5 or HDAC9 inhibitors were previously unavailable. Ecker et al. investigated the effects of the class IIa HDACis MAZ1863 and MAZ1866, which unfortunately failed to reduce metabolic activity in the medulloblastoma cell lines that were assessed [18]. In contrast, LMK-235, a novel class IIa HDACi with a preference for HDAC4 and HDAC5, displayed potent cytotoxic effects in human cancer cell lines [13]. Indeed, LMK-235 significantly attenuated cell proliferation in our study.

Moreover, LMK-235 synergized with the proteasome inhibitor bortezomib and further inhibited cell proliferation, induced apoptosis, and impeded cell invasion at lower nanomolar concentrations, which indicates that this combination therapy is more tolerable for patients. Although the potent activity of HDACis in combination with bortezomib is well documented, the underlying mechanisms remain unclear [19]. Kikuchi et al. demonstrated that the knockdown of class IIa HDAC4 enhances cytotoxicity induced by the proteasome inhibitor carfilzomib in multiple myeloma via the upregulation of activating transcription factor 4 and the activation of endoplasmic reticulum (ER) stress-induced pro-apoptotic transcription factor C/EBP homologous protein [20]. This finding provides the rationale for the combination of class IIa HDAC inhibitors and proteasome inhibitors. Due to the similarity of HDAC4 and HDAC5, we assume that HDAC5 might be involved in ER stress-induced cell apoptosis. However, further studies are warranted to verify this assumption.

In summary, our study provides compelling evidence for the important role of HDAC5 in BC progression. The clinical results showed that high HDAC5 expression correlates with inferior prognostic factors and is associated with a worse DFS. In vitro studies highlighted the tumorigenic behavior of HDAC5 in human BC cells. In addition, this finding supports the use of a promising new inhibitor, LMK-235, for inhibiting HDAC5 in BC. LMK-235 in combination with bortezomib provides a novel therapeutic strategy for the treatment of BC.

## MATERIALS AND METHODS

### Patients and tissue samples

All samples were randomly retrieved from the Department of Pathology at Fudan University Shanghai Cancer Center between January 1, 2001 and December 31, 2009. In all, 149 fresh tissues and 350 formalin-fixed paraffin-embedded (FFPE) samples fulfilled the following criteria and were selected for this study: (i) female patients diagnosed with pure primary invasive ductal carcinoma (IDC) as long as each specimen contained over 80% tumor cells. (ii) patients who had received neoadjuvant therapy were excluded. (iii) cases with reliable and reproducible results of HDAC5 RNA and protein expression were counted. Fresh tissues were frozen after surgical resection for RNA extraction. FFPE samples were constructed into TMAs for IHC. This study was approved by the independent ethical committee/institutional review board of Fudan University Shanghai Cancer Center (Shanghai Cancer Center Ethical Committee). Informed consent was remitted by the Ethics Committee.

### Quantitative real-time PCR

Total RNA extraction and cDNA synthesis were performed as described previously [21]. Briefly, RNA was extracted using an RNeasy Plus Kit (Qiagen), and reverse transcription was performed with 2 μg of total RNA. Then, the cDNA was subjected to qPCR to evaluate the relative mRNA levels of HDAC5 and GAPDH (as an internal control) in an ABI 7900HT qPCR machine (Applied Biosystems). The gene-specific primers are presented in Supplementary Table S6. Data were analyzed using the comparative threshold cycle method (2^−ΔCT^).

### Tissue microarray construction

TMAs were prepared from 450 blocks of IDC samples. The matching histological hematoxylin and eosin (HE)-stained slides were reviewed and representative areas were identified by two experienced pathologists. Two 1.0-mm-diameter cylinders of tissue were arrayed into a recipient block using a tissue microarrayer (UNITMA Instruments) as described previously [22]. In addition, 10 samples of non-tumor breast tissue were included as controls.

### Immunohistochemical staining

TMA slides were deparaffinized, rehydrated, and then incubated with rabbit polyclonal antibody against HDAC5 (Abcam) at 4°C overnight following heat-induced epitope retrieval. Staining detection was performed using a Dako LSAB+ System-HRP detection kit according to the manufacturer's instructions. An appropriate negative control was included throughout.

HDAC5 immunoreactivity was scored semiquan- titatively by two experienced pathologists who were blinded to the patients' clinical information. The H score was based on the intensity (I0, negative; I1, weak; I2, moderate; and I3, strong) and proportion (P: 0%, 5%, 10% … 100%, in 5% increments) of positively stained cells, as described previously [23]. Discordant results were resolved by a second examination by two pathologists simultaneously using a multi-headed microscope. A final H score (range, 0–300) was obtained by adding the sum of the intensity and proportion scores of the stained area (H score=[I_0_×P_0_]+[I_1_×P_1_]+[I_2_×P_2_]+[I_3_×P_3_]).

### Cell culture and treatments

BC cell lines (MCF-7, T47D, ZR-75-1, SK-BR-3, Hs-578T, MDA-MB-231, MDA-MB-436, MDA-MB-468, and MDA-MB-453), normal breast cell lines (MCF-10A and HBL-100), and HeLa cells were kindly provided by Professor ZM Shao (Fudan University Shanghai Cancer Center, Shanghai) and were cultured according to standard ATCC protocols. Cells were seeded at an approximate concentration and were cultured under standard incubation conditions (37°C, 95% humidity, 5% CO_2_) for 24 hours before the following experiments were performed: (1) transfection with sh-HDAC5 (Origene) or a scrambled control shRNA sequence, (2) treatment with LMK-235 (Selleck Chemicals) or DMSO (vehicle control), (3) treatment with bortezomib (Selleck Chemicals) or DMSO, and (4) treatment with both LMK-235 and bortezomib or DMSO.

### Western blot analysis

Equal amounts of cell lysates were subjected to 7.5% to 15% SDS–PAGE, and proteins were transferred onto PVDF membranes (Millipore). The membranes were probed overnight with specific primary antibodies, which were detected with corresponding secondary antibodies (Cell Signaling Technology). The immunoreactive bands were visualized using enhanced chemiluminescence (Thermo Scientific). The following primary antibodies were used: HDAC5 (Abcam); GAPDH (Proteintech); acetyl-histone H3, histone H3, caspase-8, caspase-9, PARP, Bim, MMP2, MMP7, and MMP9 (Cell Signaling Technology). GAPDH served as a loading control.

### shRNA transfection

HDAC5 pGFP-V-RS shRNA vectors were purchased from Origene (catalog number TG312492). Sequences of shRNA constructs are described in Supplementary Table S7. An inactive 29-mer scrambled shRNA cassette in the pGFP-V-RS vector was used as a negative control. All transfections were performed with Lipofectamine 3000 (Invitrogen) according to the manufacturer's instructions. Two (sh-HDAC5#B and sh-HDAC5#D) of four constructs with greater efficiency were selected for the following experiments.

### Cell proliferation assay

Cells were seeded and cultured in 96-well plates. A CCK8 assay (Dojindo) was performed, and the optical density (OD) at 450 nM was measured in an automatic microplate reader (BioTek). Each experiment was performed in triplicate and repeated at least twice. The data are presented as the average ±SD.

### Flow cytometry

Cells were trypsinized, centrifuged, washed twice in PBS, and stained with annexin V/PE-7AAD (BD Biosciences). Apoptosis was detected using a Cytomics FC500 flow cytometer (Beckman Coulter). The percentage of apoptotic cells was calculated using CXP Software.

### Cell invasion assay

Cells in serum-free medium (200 μl containing 3×10^4^ cells) were added to upper Transwell chambers (Corning) with an 8-mm pore size. The bottom chamber contained medium with 10% FBS as a chemoattractant. After a 24-hour incubation at 37°C, the cells on the upper surface of the membrane were removed with a cotton swab. The cells on the lower surface were fixed in ethanol and stained with 0.05% crystal violet. Cell motility was quantified by counting the number of cells that had migrated to the lower surface of the membrane. For each membrane, five random fields were counted using a light microscope at 100× magnification, and the mean value for each membrane was calculated.

### Scratch wound-healing assay

Cells were seeded in 6-well plates at a density of 15×10^5^ cells/well and incubated overnight until they reached 70% confluence. A pipette tip was used to generate a scratch in the cell layer. Cells were then transfected with shRNA (5 μg). Images were obtained at 0 and 72 hours at the same position.

### Clonogenic assay

Cells were seeded in 12-well plates and were exposed to the indicated concentration of LMK-235 for 2 to 3 weeks. Media were replaced (with drugs added) every 3 days. Cells were fixed in 4% paraformaldehyde and stained with 0.05% crystal violet.

### Statistical analysis

Statistical analyses were performed using Stata 10 statistical software. The cut off points for both mRNA and protein were selected using the area under the time-dependent receiver operating characteristic (ROC) curve by MedCalc software [24]. Patients with BC were divided into the low or high HDAC5 expression group according to the cut-off point. Differences between groups and correlations between HDAC5 expression and the clinicopathological features of patients with BC were evaluated by chi-squared tests, independent two-sample t-tests and one-way ANOVA. Survival curves were obtained using the Kaplan-Meier method with GraphPad Prism 5.0. Univariate and multivariate survival analyses were performed using the Cox proportional hazards regression model. The combination index (CI) was calculated by CompuSyn software using the Chou and Talalay method [25]. CI < 1, =1, and >1 represent synergy, additivity, and antagonism, respectively. P values less than 0.05 were considered statistically significant. Error bars represent the standard deviation unless otherwise stated.

## SUPPLEMENTARY TABLES


