# Correction: HDAC5, a potential therapeutic target and prognostic biomarker, promotes proliferation, invasion and migration in human breast cancer

**DOI:** 10.18632/oncotarget.17542

**Published:** 2017-05-01

**Authors:** Anqi Li, Zebing Liu, Ming Li, Shuling Zhou, Yan Xu, Yaoxing Xiao, Wentao Yang

**Present**: There is a duplication of images within Figure 5F and a typing error within Figure 4B

**Correct**: The proper figure images are shown below. The authors sincerely apologize for this error.

Original article: Oncotarget. 2016; 7:37966-37978. doi: 10.18632/oncotarget.9274

**Figure 4:**
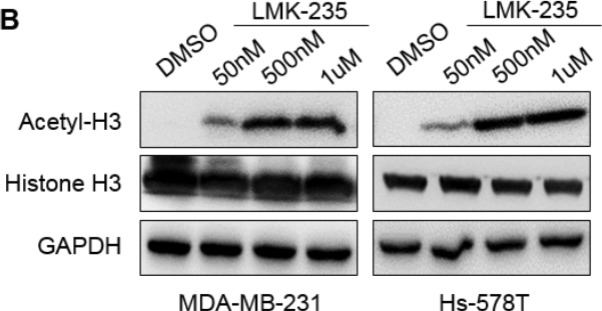
LMK-235 inhibits BC cell proliferation and induces apoptosis **B.** MDA-MB-231 and Hs-578T cells were treated with DMSO or 50 nM, 500 nM, or 1 μM LMK-235 for 24 hours. The levels of acetyl-histone H3 and total histone H3 were examined by western blot. GAPDH was used as a loading control.

**Figure 5:**
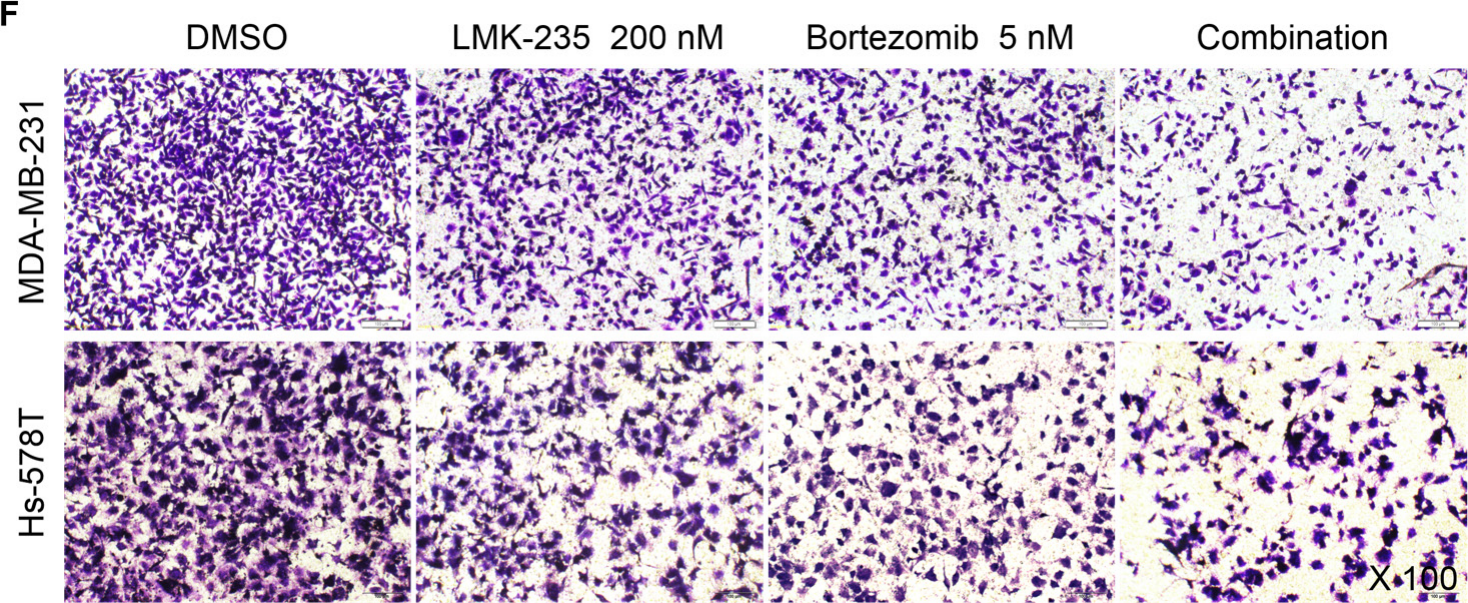
LMK-235 synergizes with bortezomib in BC cells **F.** MDA-MB-231 and Hs-578T cells were plated in Matrigel invasion chambers and treated with 200 nM LMK-235 and/or 5 nM bortezomib for 24 hours. Three separate experiments were conducted, and representative results are shown. Magnification, ×100. Columns indicate the average number of invading cells from 5 random microscopic fields. *p<0.05 compared with the DMSO group; **p<0.05 compared with the equivalent doses in the LMK-235- or bortezomib-treated groups.

