# Supplementary material for: HDAC5, a potential therapeutic target and prognostic biomarker, promotes proliferation, invasion and migration in human breast cancer
Source: Oncotarget. 2016 May 10;7(25):37966–78. doi: 10.18632/oncotarget.9274 (PMC5122364; doi:10.18632/oncotarget.9274)
Supplement: Supplementary file 1 [file oncotarget-07-37966-s001.pdf]

HDAC5, a potential therapeutic target and prognostic biomarker, promotes proliferation, invasion and migration in human breast cancer

SUPPLEMENTARY TABLES

Supplementary Table S1: Clinical and histopathologic data for 149 patients grouped by HDAC5 mRNA expression level

	Overall (N=149)		HDAC5 expression				
			Low (N=74)		High (N=75)		
	N	%	N	%	N	%	*P*
Age, years							
≤50	72	48.3%	35	47.3%	37	49.3%	0.804
>50	77	51.7%	39	52.7%	38	50.7%	
Tumor size, cm							
<2	27	18.1%	13	17.6%	14	18.7%	0.085
2≤T<5	111	74.5%	59	79.7%	52	69.3%	
≥5	11	7.4%	2	2.7%	9	12.0%	
Histological grade							
I/II	82	55.0%	35	47.3%	47	62.7%	0.059
III	67	45.0%	39	52.7%	28	37.3%	
Node status							
Negative	71	47.7%	40	54.1%	31	41.3%	0.085
Positive	76	51.0%	32	43.2%	44	58.7%	
pNX*	2	1.3%	2	2.7%	0	0.0%	
Molecular subtype							
Luminal A	50	33.6%	25	33.8%	25	33.3%	**0.009**
Luminal B	54	36.2%	19	25.7%	35	46.7%	
Her2 subtype	20	13.4%	11	14.9%	9	12.0%	
Basal-like	25	16.8%	19	25.7%	6	8.0%	
Local recurrence							
absence	142	95.3%	73	98.6%	69	92.0%	0.055
presence	7	4.7%	1	1.4%	6	8.0%	
Metastasis							
absence	124	83.2%	67	90.5%	58	77.3%	**0.028**
presence	25	16.8%	7	9.5%	17	22.7%	

*Patients with unknown LN status underwent lumpectomy or breast-conserving surgery.

Supplementary Table S2: Cox regression analysis of HDAC5 mRNA expression and clinicopathological factors predicting DFS

Variable	Univariate analysis				Multivariate analysis			
	**β**	**HR**	**95% CI**	***P***	**β**	**HR**	**95% CI**	***P***
HDAC5	0.92	2.51	1.116-5.71	**0.03**	0.84	2.33	1.00-5.30	**0.04**
Age	0.05	1.05	0.50-2.20	0.90	-	-	-	-
Tumor size	0.05	1.05	0.47-2.20	0.91	-	-	-	-
Histological grade	-0.61	0.86	0.41-1.81	0.68	-	-	-	-
Node status	0.34	1.41	1.04-1.92	**0.03**	0.32	1.37	1.00-1.87	**0.047**
Molecular subtype	0.30	1.35	0.96-1.90	0.08	-	-	-	-

Abbreviations: DFS, disease-free survival; HR, hazard ratio; CI, confidence interval.

Supplementary Table S3: Clinical and histopathologic data for 350 patients grouped by HDAC5 protein expression level

Variables	Overall (N=350)		HDAC5 expression				
			Low (N=235)		High (N=115)		
	N	%	N	%	N	%	*P*
Age, years							
≤50	179	51.14%	113	48.1%	66	57.4%	0.102
>50	171	48.86%	122	51.9%	49	42.6%	
Tumor size, cm							
<2	170	48.57%	113	48.1%	57	49.6%	0.132
2≤T<5	157	44.86%	111	47.2%	46	40.0%	
≥5	17	4.86%	8	3.4%	9	7.8%	
Unknown	6	1.71%	3	1.3%	3	2.6%	
Histological grade							
I/II	255	72.86%	169	71.9%	86	74.8%	0.571
III	95	27.14%	66	28.1%	29	25.2%	
Node status							
Negative	201	57.43%	138	58.70%	63	54.78%	0.511
Positive	147	42.00%	96	40.85%	51	44.35%	
pNX*	2	0.57%	1	0.4%	1	0.9%	
Molecular subtype							
Luminal A	86	24.57%	59	25.1%	27	23.5%	0.533
Luminal B	71	20.29%	49	20.9%	22	19.1%	
Her2 subtype	74	21.14%	53	22.6%	21	18.3%	
Basal-like	119	34.00%	74	31.5%	45	39.1%	
Local recurrence							
absence	301	86.00%	210	89.4%	91	79.1%	0.14
presence	30	8.57%	17	7.2%	13	11.3%	
Unknown	19	5.43%	8	3.4%	11	9.6%	
Metastasis							
absence	272	77.71%	197	83.8%	75	65.2%	0.001
presence	59	16.86%	30	12.8%	29	25.2%	
Unknown	19	5.43%	8	3.4%	11	9.6%	

*Patients with unknown LN status underwent lumpectomy or breast-conserving surgery.

Supplementary Table S4: Cox regression analysis of HDAC5 protein expression and clinicopathological factors predicting DFS

Variable	Univariate analysis				Multivariate analysis			
	β	HR	95% CI	*P*	β	HR	95% CI	*P*
HDAC5	0.67	1.95	1.24-3.06	0.004	0.661	1.937	1.23-3.05	0.004
Age	-0.14	0.87	0.55-1.37	0.555	-	-	-	-
Tumor size	0.25	1.29	0.87-1.90	0.204	-	-	-	-
Histological grade	0.47	1.59	1.02-2.50	0.041	-	-	-	-
Node status	0.35	1.42	1.15-1.74	0.001	0.345	1.412	1.15-1.74	0.001
Molecular subtype	0.20	1.22	1.00-1.48	0.049	-	-	-	-

Abbreviations: DFS, disease-free survival; HR, hazard ratio; CI, confidence interval.

Supplementary Table S5: The combination indexes of BC cell treated with LMK-235 and bortezomib

**MDA-MB-231**		**LMK-235 (μM)**				
		**0.075**	**0.15**	**0.3125**	**0.625**	**1.25**
Bortezomib (nM)	3.125	0.88	1.01	0.53	0.67	0.68
	6.25	0.85	0.89	0.53	0.50	0.52
	12.5	1.05	1.25	0.59	0.47	0.40
	25	0.95	0.93	0.62	0.47	0.37
	50	0.76	0.64	0.49	0.36	0.34
Hs-578T		LMK-235 (μM)				
		0.075	0.15	0.3125	0.625	1.25
Bortezomib (nM)	1.5	0.96	0.87	0.79	0.94	0.87
	3.125	0.83	1.09	1.02	0.87	0.87
	6.25	0.97	0.91	1.01	0.95	0.70
	12.5	0.83	0.77	0.71	0.56	0.47
	25	0.57	0.46	0.43	0.41	0.44

Supplementary Table S6: Information of gene-specific primers

Primer name	Sequences
GAPDH forward	TGGTCACCAGGGCTGCTT
GAPDH reverse	GTCTTCTGGGTGGCAGTGAT
HDAC5 forward	GTCTCGGCTCTGCTCAGTGTAGA
HDAC5 reverse	GGCCACTGCGTTGATGTTG

Supplementary Table S7: Information of used shRNA of HDAC5

Tube ID	Sequences	Species Specificity(human, mouse, rat)
TG312492A / GI349961	AGCACCATCGCTGAGAATGGCTTTACTGG	H
TG312492B / GI349962	GACAGTGACACCGTGTGGAATGAGATGCA	H
TG312492C / GI349963	CAACCAGTTCAGCCTCTACACGTCTCCTT	H
TG312492D / GI349964	ATGACTTGACCGCCATCTGTGATGCCTCT	H
